# The effect of deep brain stimulation on the non-motor symptoms of Parkinson’s disease: a critical review of the current evidence

**DOI:** 10.1038/npjparkd.2016.24

**Published:** 2017-01-12

**Authors:** Mónica M Kurtis, Thadshani Rajah, Luisa F Delgado, Haidar S Dafsari

**Affiliations:** 1 Movement Disorders Unit, Neurology Department, Hospital Ruber Internacional, Madrid, Spain; 2 Kings Parkinson's Centre of Excellence, Kings College and Kings College Hospital, London, UK; 3 Fundación Universitaria de Ciencias de la Salud, Hospital San José—Hospital Infantil Universitario de San José, Bogotá, Colombia; 4 Department of Neurology, University Hospital Cologne, Cologne, Germany

## Abstract

The benefit of deep brain stimulation (DBS) in controlling the motor symptoms of Parkinson’s disease is well established, however, the impact on the non-motor symptoms (NMS) remains to be elucidated, although the growing investigative efforts are promising. This article reviews the reported data and considers the level of evidence available with regard to the effect of DBS on NMS total burden and on the cognitive, neuropsychiatric, sleep, pain, dysautonomic, and weight domains. Multiple case series suggest that DBS improves the burden of NMS by reducing prevalence, intensity, and non-motor fluctuations. There is level I evidence on the effect of DBS on cognition and mood. Slight cognitive decline has been reported in most class I studies, although the functional effect is probably minimal. Two randomized prospective studies reported no change in depression while improvement of anxiety has been reported by a class I trial. Prospective cohort studies point to improvement of hyperdopaminergic behaviors, such as impulse control disorders, while others report that hypodopaminergic states, like apathy, can appear after DBS. There is only class III evidence supporting the benefit of DBS on other NMS such as nocturnal sleep, pain, dysautonomia (urinary, gastrointestinal, cardiovascular, and sweating), and weight loss. Although preliminary results are promising, randomized prospectively controlled trials with NMS as primary end points are necessary to further explore the effect of DBS on these often invalidating symptoms and offer conclusions about efficacy.

## Introduction

The efficacy of deep brain stimulation (DBS) on the control of motor symptoms in Parkinson’s disease (PD) has been consistently demonstrated in Class I clinical trials and is thus well established.^1–3^ In the past two decades there is a growing recognition that non-motor symptoms (NMS) are fundamental to the concept of PD as they dominate the premotor stage and are prevalent throughout disease progression.^4^ A single NMS, like pain or depression, can overshadow the clinical picture in some patients, and NMS burden (NMSB) as a whole has been recently identified as the most important factor in determining the quality of life of PD patients.^5^ The importance of NMS makes the need for effective therapies increasingly evident and thus behooves the scientific community to investigate the effect of DBS on these symptoms. There are previous reviews concerning this topic,^6–8^ but the need for an updated critical review of the current data is warranted as the number of publications regarding NMS and DBS has grown exponentially in the past years. To give the reader an analytical overview, studies are reviewed in depth and classified according to the quality of evidence they provide: level I (large randomized controlled trials and meta-analysis), level II (small randomized trials and controlled prospective trials), and level III (prospective uncontrolled case series), following parameters established by the evidence-based task force commissioned by the Movement Disorders Society.^9^

This review includes clinical studies considering NMS as a primary or secondary outcome from DBS intervention and focusing on NMSB, non-motor fluctuations (NMF), cognition, neuropsychiatric symptoms (depression and anxiety, suicide attempts, apathy, and impulse control disorders (ICDs)), sleep, pain, autonomic (urinary, gastrointestinal, cardiovascular, and sweating), and weight gain.

## The pathopysiology of NMS and DBS

The pathophysiology underlying cognitive and neuropsychiatric manifestations in PD is certainly complex and individually variable. Multiples studies corroborate widespread Lewy type-α synucleinopathy (LTS) in the neocortex as an important factor, and limbic and brainstem involvement may also have a role, as well as coexisting Alzheimer’s disease pathology, cerebrovascular disease, and amyloid angiopathy.^10^ The implication of cortico-basal ganglia (BG) circuitry disorders in the development of these NMS is also gaining attention as understanding of these networks progresses.

On the basis of the model developed by Alexander *et al.*^11^ in 1986, it is generally accepted that the BG constitute a part of three distinct functional and anatomical loops involving sensorimotor, associative, and limbic processing.

These networks have a role in weeding out relevant information from noise for the selection of movement patterns, actions, and goal-directed behaviors, while neurophysiological and neuroimaging studies have delineated their segregated topographical organization into parallel circuits. It is the ventral striatum and caudate output pathways that project, through the ventromedial globus pallidus, subthalamic nucleus (STN), substantia nigra, and thalamus, to the anterior cingulate and dorsolateral prefrontal and lateral orbitofrontal cortices (which send return pathways to these structures) thus regulating cognitive and behavioral processes. The well-known motor circuit involves the direct and indirect pathways and connects the dorsolateral putamen, globus pallidus interna (GPi), STN, and thalamus to the motor and premotor cortical areas.^12,13^ The dopamine depletion seen in PD leads to a reduction of selectivity and spacial focalization of this cortico-BG circuitry and thus may be the pathological substrate of, not only motor symptoms such as brdykinesia and rigidity, but also non-motor symptoms such as apathy and dysexecutive functions.

Dopamine replacement therapy may restores the balance. However, because striatal dopamine depletion is heterogeneous, treatment may lead to hypersensitivity of postsynaptic dopamine receptors in selective subterritories of the striatum. If the dorsolateral striatal territory expresses this sensitization, the result can be augmented movements (dyskinesias). If more ventromedial regions are implicated, ICDs may arise.^12^ It is well established that DBS surgery in the main relay centers of the cortico-BG circuitry, such as STN or GPi, has similar clinical effects to dopaminergic therapy. It is thought that DBS high frequency stimulation in the dorsolateral region of the STN facilitates movement by releasing the ‘No Go’ signal normally exerted by this nucleus on the motor BG circuitry. It is possible that an anteromedial electrode placement or current spread to this area may also release the ‘No Go’ signal on the limbic and associative circuits^13^ and thus produce cognitive and affective disinhibition, explaining some of the neuropsychiatric NMS effects seen after DBS.^14,15^

The pathophysiological basis for DBS effect on other non-motor symptoms such as sleep disorders, pain, and dysautonomia is complex and different mechanisms may have a role. First, adjacent regions near the STN, for example, the pedunculopontine nucleus, could be modulated by DBS, thus resulting in beneficial effects on sleep.^16^ Second, a modulation of BG circuitry may result in effects on autonomic centers of the thalamus, projecting to the anterior cingulate and lateral frontal cortex with beneficial effects of symptoms like sweating^17^ and bladder control.^18^

## DBS effect of NMSB as a whole

NMSB can be evaluated by the NMS Questionnaire (NMSQuest),^19^ a qualitative patient completed tool, or measured in a quantitative fashion by the NMS Scale (NMSS).^20^ However, there have only been a few studies that have specifically used validated tools for measuring NMS in relation to DBS in PD (see Table 1). In a small open-label study including 10 PD patients, a 36% overall improvement in the mean NMSS score after STN DBS surgery was reported.^21^ However, the sample was too small to be able to report any meaningful subgroup/domain analysis. Using the NMSQuest-based scoring system, Nazzarro *et al*.^22^ studied 24 patients who underwent bilateral STN DBS and showed that the NMSQuest score decreased from 12 (severe NMSB) pre-surgery to 7 (moderate NMSB) post surgery at 1-year follow-up.^22^ Subsequently, Dafsari *et al.*^23^ have reported the first multicentre European DBS study of STN stimulation where NMS were the primary outcome.^23^ This was an open prospective observational study including 60 patients who underwent STN DBS and were evaluated at baseline and at 6-month follow-up with the NMSS. The authors reported a 42% improvement in NMSB, underpinned by a range of significant improvements in specific NMS (sleep, urinary, and perceptual problems). The immediate effect of DBS on NMSB has also been studied with nonspecific tools. Wolz *et al*.^24^ reported on 10 NMS in 34 patients with a median DBS time of 13 months. In this cohort, DBS did not have an immediate effect on the frequencies of a wide range of autonomic, sensory, cognitive, and neuropsychiatric NMS as assessed by a semi-structured interview, and only a significant improvement of the frequency of ‘inner restlessness’ was reported.^24^ However, the severity of most NMS, as measured by Visual Analog Scales, was significantly improved; particularly fatigue, and inner restlessness in a subset of patients. The methodological differences in these studies including objectives, measuring tools, and follow-up times may account for the variability of results.

## DBS effect on non-motor fluctuations

The NMF accompany motor fluctuations in most cases and are common in PD.^25^ NMF may exist as NMS symptoms that typically worsen during motor-off periods, that are only present during motor-off periods, or that fluctuate independently from the motor state.^25^ Witjas *et al.*^26^ studied NMF in 40 patients who underwent STN DBS surgery and were followed for 1 year.^26^ They classified NMF into four main groups: cognitive, psychiatric, autonomic, and sensory fluctuations. The NMF that improved the most after STN DBS were sensory symptoms and pain, with 84.2% improvement. Other NMF which improved were in the dysautonomic and cognitive domains with 60 and 70% decrease in severity. However, psychiatric fluctuations seemed to respond less consistently postoperatively. Ortega-Cubero *et al.*^27^ studied 20 patients and reported reduced frequency and severity after 2-year follow-up of both psychiatric and autonomic fluctuations after STN DBS surgery, partially supporting the previous observations.^27^ These open-label data (see Table 1) implying the benefit of DBS on NMSB and NMF need to be confirmed in controlled studies. These small sample studies probably included subjects with a range of baseline characteristics regarding their NMF that may explain the differing results. For example, pre-surgical NMF response to levodopa therapy, duration, severity, and frequency may influence response to DBS.

## DBS effect on cognition

Cognitive decline is far-reaching in PD and seems an inevitable consequence of disease progression as more than 80% of patients develop dementia after 20-year follow-up.^28^ A large meta-analysis including more than 600 patients showed that DBS of the STN generates a statistically significant small decrease in the executive functions and working memory.^29^ Similarly, a class I study by Witt *et al.*^30^ found mild decrease in executive functions in the STN group when compared with the best medical therapy group (BMT) after a 6-month follow-up.^30^ However, another subsequent meta-analysis including more than 10 000 patients reported that 57% of the included studies did not find significant cognitive changes after DBS.^31^ This was corroborated by a large randomized prospective study by Willams *et al.*^32^ comparing a BMT group with an STN surgery group during a 1-year follow-up, and showing no differences in cognitive decline.^32^ Other prospective uncontrolled studies have found none or clinically irrelevant cognitive decline,^33–37^ while some report a decline in lexical fluency.^33,36–38^ Two large randomized studies compared the classic surgical targets for PD and showed no difference between the groups.^3,39^

These variable results may be partly attributed to patient cohort differences with respect to baseline demographic characteristics, PD symptomatology, surgical techniques, and postoperative management. The main risk factors associated with cognitive decline and surgery have been subdivided into (1) preoperative risk factors: impaired attention, higher levodopa equivalent dosages and motor impairment, older age, higher motor, and severe axial symptoms; (2) intra-operative risk factors such as surgical electrode trajectories (through the frontal lobe and head of the caudate nucleus); and (3) postoperative factors including stimulation parameters, electrode location, and medication changes.^14,40^

In conclusion, there is level I and II type evidence (see Table 2) consistently reporting a moderate decrease in verbal fluency after STN DBS, as well as a mild reduction in abstract reasoning, working memory and executive functions. However, this mild cognitive deterioration is generally not clinically relevant.

## Dbs effect on neuropsychiatric symptoms

### Depression

Patients with PD may manifest neuropsychiatric symptoms such as depression, anxiety, apathy, impulsiveness, introversion, hopelessness, and suicidal behavior at any stage of the disease. Depression is considered one of the most frequent mood state changes in PD with a prevalence of about 40–50%, with major depression occurring in 5 to 10% of cases.^8^ Two large randomized controlled trials showed that there was no difference between the STN DBS and BMT groups with respect to depression after a 6-month follow-up (See Table 2).^1,2^ One randomized study comparing the difference in depressive symptoms between the two classic targets, did not show differences,^39^ while Follet *et al.*^3^ reported worsening of depression after STN DBS when compared with GPi.^3^ Smaller controlled trials on the immediate effect of DBS^41^ and prospective case series with up to 11-year follow-up have reported no change in depression,^38,42^ or mild improvement.^37,43^ In summary, there is level I evidence demonstrating that DBS is not detrimental for depression, and some class II and III studies suggesting it may improve slightly. Differing results may be due to population differences, methodological variations (i.e., measuring tools, follow-up time) and postoperative motor control and medical treatment changes.

### Anxiety

Anxiety is another frequent NMS, usually coexisting with depression and with motor fluctuations, occurring in about 40% of PD patients.^8^ There is one large randomized controlled study including 156 patients who underwent STN DBS or BMT providing class I data of improvement in anxiety scales at 6-month follow-up after DBS (see Table 2).^30^ Similarly to depression results, some prospective series suggest that anxiety may benefit from STN DBS.^41,43^

### Impulse control disorders

ICDs are behavioral addictions that include hypersexuality, hyperphagia, pathologic gambling and compulsive shopping. These ICDs affect up to 40% of PD patients with dopamine agonist therapy and approximately 15% of PD patients overall.^44^ There are conflictive results as far as their response to surgery (see Table 3). One of the recognized causes of ICDs relates to a hyperdopaminergic state, related to dopamine replacement therapy. Various large prospective studies suggest that when ICDs are caused by hyperdopaminergic states, they improve after surgery due to drug reduction.^43,45,46^ Lhommée *et al.*^43^ go as far as proposing that disabling dopaminergic treatment abuse and drug-induced behavioral addictions in PD may be considered a new indication for subthalamic stimulation.^43^ However, the verdict is still not settled since some cases of *de novo* impulsivity have been reported after surgery.^47–49^ In a large retrospective study including 89 patients, Kim *et al.*^49^ reported improvement in 13 of 20 patients with ICDs, however nine cases developed *de novo* ICDs.^49^ In a survey directed to the Parkinson Study Group centers with the objective of evaluating the prevalence and screening of ICDs pre and post DBS, 67% of the centers observed at least one case of *de novo* ICD occurrence after surgery.^50^ These seemingly contradictory results may be due to differing underlying pathophysiological mechanisms. ICDs that are related to a hyperdopaminergic state clearly improve with medication adjustments after DBS. However, in a subset of patients with greater mesolimbic degeneration, DBS itself may produce alterations in the limbic BG-loop and lead to hyperactivation of the ventral striatum and development of ICDs.

### Apathy

Apathy is a behavioral disorder characterized by decreased motivation, emotional involvement, and goal-directed and self-driven behaviors. It has been described in about 40% of patients with PD^51^ and has recently been classified into four subtypes relating to a (1) reward deficiency syndrome, (2) emotional distress, (3) executive dysfunction, and (4) autoactivation deficit.^52^ Improvement in apathy has been reported after starting dopaminergic therapy and after turning on STN DBS.^41^ However, worsening of apathy after surgery has also been reported, more than a decade ago,^36^ and in a more recent prospective study including 63 patients, Thobois *et al.*^51^ described how 34 patients developed apathy in the first months (mean 4.7 months, 3.3–8.2 months) post surgery (see Table 3). In this seminal study, the authors related the *de novo* apathy cases to a rapid withdrawal of dopaminergic drugs after surgery. However, medication changes do not explain the whole story in postsurgical apathy and pre-existing risk factors such as greater dopaminergic mesolimbic degeneration,^12^ NMF,^51^ and dyskinesias may have a role.^53^ Similar to the post-DBS results for ICDs, these seemingly contradictory results probably depend on the subtype of apathy and its pathophysiological basis.

### Suicide risk

The increased risk of suicidal behaviors after DBS remains an open-ended question. Attention was drawn to this subject by a large meta-analysis reviewing the first decade of DBS results, which described a completed suicide rate of 0.16–0.32% after DBS.^31^ This issue was further explored by Voon *et al.*^54^ in a multicenter retrospective case series, including 5311 patients, that reported an attempted suicide rate of 0.9% and a completed suicide rate of 0.45% in the first postoperative.^54^ These alarming results were not corroborated in a later randomized controlled trial by Weintraub and colleagues^55^ comparing a BMT group including 299 patients and a DBS group (including both STN and GPi targets) of 255 patients.^55^ However, this trial was possibly underpowered owing to the rarity of suicidal events. The risk factors for suicidal ideation and behaviors proposed by this group included selection bias of personality traits in surgical patients, depression, previous history of ICDs, and unrealistic expectations of surgery.

## DBS effect on sleep

PD patients commonly report sleep problems, suffering from insomnia, excessive daytime sleepiness, restless legs syndrome, and REM sleep behavior disorder (RBD).^56,57^ Several studies have investigated the effect of DBS on sleep dysfunction in PD (see Table 4). Iranzo and colleagues^58^ were pioneers in showing objective improvement in sleep quality studied by polysomnography after STN DBS. They reported increased continuous sleep time and a decreased arousal index in 11 patients at 6-month follow-up.^58^ Hjort *et al.*^59^ reported improvement of overall sleep quality after STN DBS as they found significant improvement in the mean total Parkinson Disease Sleep Scale after 3 months.^59^ They did not observe change in nocturia or excessive daytime sleepiness despite medication reduction. In another study in a larger cohort, Amara *et al.*^60^ reported subjective sleep quality improvement after unilateral STN DBS as measured by the Pittsburgh Sleep Quality Index.^60^ Dafsari *et al*.^23^ also provide significant evidence of subjective sleep improvement after DBS.^23^ The long-term benefit of sleep after DBS is supported by an early study by Lyons and Pahwa^61^ that found consistent improvement for up to 2 years.^61^ As previously reported, this group did not find change in excessive daytime sleepiness.^61^ In summary, there is level III type evidence suggesting that STN DBS may improve nocturnal sleep in PD patients, particularly sleep quality. Most authors hypothesize that sleep benefit after DBS could result from improved nocturnal mobility and reduced sleep fragmentation. Current data do not report any reduction in REM sleep behavior disorder or improvement of excessive daytime sleepiness.

## DBS effect on pain

There have been several open label studies addressing the issue of pain, a common NMS of PD and its response to DBS, using either STN or the GPi as targets (see Table 4). Loher *et al.*^62^ reported on the effect of unilateral and bilateral GPi DBS in 16 patients: evaluations documented at 3 months indicated pain relief, which was sustained at the 12-month follow-up.^62^ In another study, Kim *et al.*^63^ reported a positive outcome of pain after STN DBS that persisted after 2 years, although some patients developed *de novo* musculoskeletal pain.^63^ In their recently published study, the IPMDS NMS study group showed that after 6 months of STN DBS, there was significant reduction in pain scores as measured by the NMSS.^23^ It is worth noting that these studies do not specifically address the classification of pain in PD. On the other hand, Cury *et al.*^64^ investigated a cohort of 41 patients and tackled this issue specifically, reporting significant improvement in pain intensity after 1 year of STN DBS.^64^ The prevalence of pain decreased from 70 to 21% and the most improved types were dystonic and musculoskeletal pain while central and neuropathic pain did not change. This supports previous findings with quantitative sensory testing describing that sensitivity to pain was not influenced by STN DBS and suggesting that DBS may have no direct modulation of central pain processing.^65^

Pain and DBS studies to date suffer from methodological limitations due to their open label nature and lack of a coherent tool to address the multifactorial nature of pain in PD that is currently possible with the recently validated King’s PD Pain Scale.^66^ There is only class III evidence supporting the benefit of DBS on pain and future randomized controlled studies should take into account the various types of PD-related pain as they may be influenced differently by DBS.

## DBS effect on the autonomic domain

### Urinary symptoms

Urinary symptoms are important constituents of autonomic dysfunction and occur in 38–71% of PD patients and are therefore of the most frequent NMS.^57^ Another review has reported improvement in detrusor hyperreflexia and increased bladder capacity after DBS.^7^ A recent study reported significant improvement in urinary symptoms from baseline in the urinary domain of the NMSS, which addresses frequency, urgency, and nocturia.^23^ In other open label studies studying the immediate effects of turning DBS ON, Seif *et al.*^67^ found improved bladder function as measured by objective urodynamic parameters^67^ and Wolz *et al.*^24^ found that patients reported a positive direct effect on urinary symptoms with DBS on.^24^ The underlying physiological mechanism may be related to improved cortical control,^68^ and improved sensory gating.^18^ However, these data need to be corroborated in further larger studies as some have reported subjective benefit but no change in urodynamic parameters (see Table 5).^69^

### Gastrointestinal symptoms

Not many studies have specifically looked at the effect of DBS on the gastrointestinal system (see Table 5). The first study to document evidence was conducted by Arai *et al*.^70^ who studied 16 PD patients and measured gastric emptying by the excretion of CO_2_ in the ^13^C-acetate breath test.^70^ They reported improvement of gastric emptying after turning DBS on and speculated on a modulatory role of the dysfunctional vagal neural control in PD. The study by Dafsari *et al.*^23^ supports the above-mentioned findings of improved gastrointestinal emptying after STN DBS.^23^ Another study by Zibetti *et al.*^71^ reported the positive effects of STN DBS in improving salivation and constipation in a 1- and 3-year follow-up of 36 patients.^71^ There is therefore class III evidence suggesting some benefit in gastrointestinal symptoms after DBS. Future studies should take into account the complexity of underlying pathological mechanisms. For example, constipation may depend on a central component (mediated by neural loss and LTS in the dorsal motor nucleus of the vagus nerve and spinal cord) and a peripheral component (mediated by a rostral–caudal gastrointestinal gradient of LTS). Patients with more brainstem pathology, may obtain a benefit not seen in others.

Troche *et al.*^72^ reviewed nine studies that specifically addressed the effect of STN DBS on dysphagia and found no effect.^72^ However, in a recent manometric study in 16 PD patients, Derrey *et al.*^73^ found significant improvement in esophageal body contractions and enhancement of lower esophageal sphincter opening. Again, dysphagia in PD is a complex symptom that may be caused by alterations in the oral, pharyngeal, or esophageal phases of swallowing. On the basis of a small series of autopsy studies, dysphagia probably depends on LTS pathology of the pharyngeal nerves and localized muscle atrophy^10^ and thus one would not expect to see an effect by DBS.^10^ However, some patients may also show abnormal esophageal peristaltic movements, which could be modulated by DBS through the vagal nerve, but whether this translates into clinical benefit remains to be elucidated.^74^

### Orthostatic hypotension

An early study in a small cohort indicated instant improvement of orthostatic hypotension after turning STN DBS on,^75^ but failed to report any change in cardiovascular control (see Table 5). These findings were supported by a more recent controlled study that reported improved orthostatic hypotension in PD patients after DBS.^76^ Another controlled study including 28 PD patients proposed that the vagal component of the arterial-cardiac baroreflex improved postoperatively.^77^ Dafsari *et al*.^23^ suggested that STN DBS may have a long-term effect on cardiovascular symptoms, and opine that further studies are needed.^23^

### Hyperhidrosis

There have been contradictory outcomes on the effect of STN DBS on sweating function, where some studies show positive results and others fail to observe differences (see Table 5). Wolz *et al.*^24^ found no change after immediate stimulation;^24^ whereas Dafsari *et al.*^23^ found significant improvement in hyperhidrosis at 6-month follow-up.^23^ In another 6-month study including 19 patients, Trachani *et al.*^17^ indicated that there was subjective improvement in sweating, however, no objective reduction in hyperhidrosis was demonstrated.^17^ These studies were limited by the small number of included subjects, and offer class III evidence that DBS probably does not worsen sweating spells. Contradictory results can partially be explained by differences in baseline characteristics (e.g., sweating spells clearly related with dyskinesias, off periods, or nighttime akinesia may benefit from DBS due to motor improvement), measuring tools (there is no validated tool that takes into account sweating fluctuations, subjective perception, and objective findings) and postsurgical medication changes.

## Olfaction

Although odor detection^78^ and identification^79^ seems to not be influenced by DBS, odor discrimination seems to be improved as a result of DBS,^78^ possibly owing to improved cognitive odor information processing.^80^ In a recent open-label, prospective study including 60 patients with PD, patients experienced a subjective improvement of the ability to smell and taste.^23^

## DBS effect on body weight

Generally, patients with PD lose weight gradually as the disease progresses.^81^ The effect of DBS on body weight is still a matter under discussion; however, in recent years, many observational studies (see Table 6) have reported that patients gain weight after surgery and this may adversely affect their metabolic status.^82^ In a retrospective review including 182 patients with a range of movement disorders and DBS targets, Strowd *et al.*^83^ described a mean weight gain of about 1 kg per year for 2 years following surgery.^83^ In a recent retrospective case–control study, this same group reported a mean weight gain of 2.9 kg in the STN DBS group, significantly more than in the control group on BMT, with mean loss of 1.8 kg.^84^ An early prospective study showed a 4.7 kg. increase in PD patients after surgery at 1-year follow-up.^85^ Other groups have reported weight gain ranging from 4.3 to 14 kg.^86^ One study explored this weight gain by gender and found that men gained lean body mass, whereas women gained weight at the expense of fat, but mean weight gain was similar.^87^ Another study evaluated the difference between STN DBS patients and BMT, showing greater weight gain in the surgical group.^88^ There are different hypotheses attempting to explain postsurgical weight gain, relating it to dopaminergic therapy modifications, increased food intake related to changes in the hypothalamus,^89^ decreased energy expenditure by better control of dyskinesias,^90^ and improvement of motor symptoms.^85^ In summary, there is Class III evidence suggesting that PD patients gain weight after STN DBS.

## Discussion

This review of the current data investigating the impact of DBS on the NMS suggests that some symptoms may improve, others remain unchanged and some worsen, corroborating previous findings.^6–8^ There is high-quality evidence demonstrating that DBS is generally a safe procedure with regard to cognition and behavioral morbidity. Data support the improvement of anxiety, and possibly also depression and ICDs. The benefit on sleep, dystonic, and musculoskeletal pain, urinary and gastrointestinal symptoms, and weight loss has also been suggested by prospective uncontrolled studies. Overall, cognitive function generally remains stable, while some small studies suggest that REM sleep behavior disorder, excessive daytime sleepiness, and dysphagia also remain unchanged by DBS. On the other hand, after surgery, decreased verbal fluency is consistently reported and apathy possibly worsens, as suggested by prospective series.

In interpreting postsurgical results, the reader must bear in mind the limitations of each type of study (Table 1,2,3,4,5,6). Even in high-quality studies, it is difficult to extrapolate the direct impact of DBS from the possible indirect effects that may be modulated by changes in medication, motor symptom improvement, or management of therapeutic expectations, among many other variables.

Substantial progress has been made in recent years to further our understanding of the NMS. Development of disease-specific patient-reported questionnaires, such as the NMSQuest^19^ or questionnaire for impulsive-compulsive disorders in Parkinson's disease,^91^ and disease-specific scales, like the NMSS,^20^ the REM Sleep Behavior Disorder Severity Scale,^92^ and King’s PD Pain Scale,^66^ have provided the necessary tools to measure NMS, thus enabling an increasing number of clinical trials to include specific NMS and NMS as a whole as primary end points. DBS multidisciplinary investigative efforts combining functional neuroimaging, neurophysiology, and clinical data provide a unique opportunity in advancing toward understanding the neurophysiological mechanisms behind NMS. A key point in the future may be to understand how the somatotopy of the BG loops influences various non-motor outcomes. Neuropsychiatric and cognitive results probably depend more highly on cortico-BG circuitry than other non-motor symptoms, such as pain and dysautonomia, that may have a central and peripheral component, as suggested by neuropathologic findings of LTS at both levels.^10^

Of the classic DBS targets, most of the data pertains to the subthalamic nucleus (STN), with a paucity of studies regarding the GPi, and null investigations specifically looking at the effect of thalamic ventroimtermediate stimulation. There are few comparative studies available for non-motor effects of STN and GPi DBS. Although some data suggest, advantages of GPi DBS on mood and behavior,^55,93,94^ little is known on other non-motor symptoms such as autonomic dysfunctions, pain or sleep symptoms. Future studies comparing STN and GPi DBS effects should not only focus on the quality of life and motor symptoms but also on a wide range of non-motor symptoms. This requires the utilization of scores covering a wide range of NMS such as the NMS Scale, the NMS Questionnaire, and laboratory-assisted evaluation of NMS with, e.g., cognitive tests such as the verbal fluency for executive dysfunctions, polysomnography for sleep, CO_2_ excretion measurement for gastrointestinal functions, urometric tests for urinary symptoms, etc.

DBS was initially developed to target medical refractory motor symptoms such as severe dyskinesias and fluctuations. However, we have since learned that NMS in PD may affect patient quality of life to a higher degree than motor symptoms^5^ and current effective medical treatments are lacking. Recent DBS technology developments have provided directional stimulation systems (Boston Scientific and Medtronic development center Eindhoven), which allow current steering towards specific subareas of anatomic target structures. Case reports^95^ and smaller scope^96^ studies pioneering this technology in patients with PD have provided evidence of its clinical usefulness to achieve optimized motor effects and avoid side effects of DBS. On the basis of the anatomy and functional circuitry of the BG, there is a rationale to examine the non-motor effects of DBS in subareas of the target region in a similar experimental design as in these studies on motor effects of directional stimulation.

We advocate that future randomized controlled studies in DBS should (1) include NMS as primary end points, (2) involve large cohorts that can be divided into subtypes,^97^ and (3) specifically analyze volumes of tissue activated in context of patients’ individual BG anatomy/somatotopy and thus advance towards therapies tailored personally, based on motor and non-motor symptom profile.

## Figures and Tables

**Table 6:** DBS effects on body weight in patients with PD

*Author*	*Year*	*Outcome measure*	*Type of study*	*No. of patients*	*Main outcome after DBS*
Strowd *et al.*^84^	2016	Height, weight and BMI	2 years retrospective case–control	69; 35 STN (10 uni, 25 bil) 34 BMT	STN-DBS group: WG mean 2.9 kg versus BMT group mean *W* loss of 1.8 kg (1.82±6.9 kg). No difference between uni and bil STN.
Strowd *et al.*^83^	2010	Weight	2 years retrospective DBS for PD, ET, dystonia	182	Mean WG of 1.8 kg at 24 month follow-up in the whole sample. In the PD subpopulation, mean WG of 2.3 kg.
Walker *et al.*^88^	2009	Weight	1-year case–control	39 uni STN 40 BMT	STN DBS (case) mean WG of 4.3 kg compared with controls (BMT).
Montaurier *et al.*^87^	2007	Body composition Dual X-ray absorptiometry and energy expenditure were measured over 36 h in calorimetric chambers.	4 months prospective open	24	Normalization of energy metabolism after DBS-STN implantation may favor body WG. Men gained primarily fat-free mass. Women gained mainly fat mass.
Macia *et al.*^90^	2004	Weight and BMI	Case–control	19 STN 14 BMT	The SNT-DBS group had a significant weight gain (9.7±7 kg) and BMI increase (4.7 kg/m^2^) vs. control group.
Gironell *et al.*^85^	2002	Questionnaire about severity and etiology of weight again	1-year prospective	27	Mean WG was 4.7 kg after surgery. Significant correlation between WG and improvement of dyskinesias. Majority of patients referred WG as adverse event.

Abbreviations: BC, body composition; bil, bilateral; BMI, body mass index; BMT, basal metabolic rate; DBS, deep brain stimulation; ET, essential tremor; PD, Parkinson’s disease; STN, subthalamic nucleus; uni, unilateral; *W*, weight; WG, weight gain.

**Table 2:** DBS effects on cognitive impairment and mood in patients with PD

*Author*	*Year*	*Outcome measure*	*Type of study*	*No. of patients*	*Results after DBS*
Rizzone *et al.*^38^	2014	Secondary: cognition and mood MMSE, RPM, digit span forward, phonological fluency, Corsi’s block-tapping test forward/backward MWCST, RAVLT, SAS, SDS, BDI and STAI	11-year prospective multicenter	26 STN	Remarkable decline in phonological fluency, slight decline in cognition. No significant changes in depression. Contradictory results in anxiety.
Oderkerken *et al.*^39^	2013	Primary: cognition and mood Composite score (cognitive tests, loss of professional activity, work, or job; loss of important relationships; psychosis, depression, or anxiety for 3 months or longer as by MINI)	1-year randomized controlled trial	128 (63 GPi) (65 STN)	No statistically significant difference between GPi and STN groups.
Lhommée *et al.*^43^ See ref. 51	2012	Secondary: cognition mood, behavior and NMF. MDRS, Frontal Score (MWCST, verbal fluency, graphic and motor series) MINI, Ardouin Scale, BDI, BAI and SAS^1^	1-year prospective Multicenter	63 STN	Overall cognition unchanged. Reduction in verbal fluency. Improvement in MWCST. Depression and anxiety improved.
McDonald *et al.*^41^	2012	Primary: mood VAMS	Cross-sectional On/off DBS	34 (23 STN) (11 BMT)	Mood, anxiety, apathy and fatigue improve with STN DBS ON.
Follett *et al.*^3^	2010	Secondary: cognition and mood WAIS III and BDI-II	2-year randomized prospective multicenter	299 (152 GPi) (147 STN)	Similarly slight decrements in cognitive function in STN and GPi DBS group. Depression worsened after SNT-DBS and improved after GPi DBS.
Williams *et al.*^32^	2010	Secondary: cognition DRS-II, D-KEFS and WAIS	1-year randomized prospective open-label	366 (182 STN) (183 BMT)	No difference between STN-DBS and BMT groups in DRS-II scores. Small significant decrease in verbal fluency in STN versus BMT.
Weaver *et al.*^2^	2009	Secondary: cognition and mood MDRS, WAIS, verbal associative fluency, Stroop Test, WCST, Boston Naming Test, Brief Visuospatial Memory Test, Hopkins Verbal Learning Test. BDI	6 months randomized controlled	255 (60 STN) (61 GPi) (134 BMT)	Neither treatment was associated with significant change in cognition or depression.
Witt *et al.*^30^	2008	Primary: cognition MDRS, RAVLT (German), WAIS Benton visual retention, stroop test, verbal fluency	6 months randomized multicentre	123 (60STN) (63BMT)	STN DBS does not reduce overall cognition. Selective decrease in frontal cognitive functions. Anxiety improves in the STN group compared to BMT group.
Kaiser *et al.*^42^	2008	Primary: Mood and psychosocial function POMS, BDI, STAI-X1/X2, SCL-90-R and SIP	3-year prospective	33 STN	No change in mood or psychosocial functioning. A cluster analysis revealed 4 different psychosocial profiles that remain stable after surgery.
Appleby *et al.*^31^	2007	Secondary: cognition, depression, behavior Neuropsychiatric tests, psychiatric side effects	Meta-analysis 546 articles published between 1996–2005	10,339 DBS	Reported rates of depression, cognitive impairment, mania, and behavior changes are low. High rate of suicide in patients treated with DBS.
Deuschl *et al.*^1^	2006	Secondary: cognition and mood MDRS, Montgomery and Asberg DRS, Brief Psychiatric Rating Scale	6-month randomized-pairs trial	156 78 STN 78 BMT	Emotional and cognitive measures did not differ significantly.
Parsons *et al.*^29^	2006	Primary: cognition Neuropsychiatric tests	Meta-analysis 20 articles published between 1990–2005	612 STN	STN-DBS results in slight decrease in executive functions and working memory.
Castelli *et al.*^37^	2006	Primary: Cognition, mood, psych disorders RCM, Bisyllabic Word Repetition Test, Corsi's Block-Tapping Test, Paired-Associate Learning, Trail Making-B, NMCST, verbal fluency tasks,BDI, STAI and SCID-II	15-month prospective	72 STN	No overall cognitive change. Mild decrease in verbal fluency. Small improvement in depression and obsessive-compulsive and paranoid personality traits.
Funkiewitz *et al.*^36^	2004	Primary: cognition, mood and behavior MDRS, WCST, verbal fluency graphic and motor series, BDI, UPDRS I	3-year prospective	77 STN	No global cognitive deterioration. Verbal fluency decline.
Krack *et al.*^34^	2003	Secondary: cognition MDRS, BDI, Clinical interview	5-year prospective	49 STN	Cognitive function changes between year 1–5 are consistent with progression of PD.
Daniele *et al.*^35^	2003	Primary: cognitive MMSE and MWCST Corsi’s block-tapping test, RAVLT Clinical Interview	12-month prospective	20 STN	No change in global cognitive function.
Pillon B *et al.*^33^	2000	Primary: cognition MDRS, Grober and Buschke, MWCST, Stroop test, trail making, verbal fluency, CANTAB BDI	1-year prospective multicenter+cross-sectional (off/on DBS)	56 (48-STN) (8-GPi) 20 (15 STN) (5 GPi)	STN patients had no cognitive deficit at 12 months, except for lexical fluency. There was no differential effect of STN or GPi stimulation. With STN DBS on: mild but significant improvement in psychomotor speed and working memory.

Abbreviations: BAI, Beck Anxiety Inventory; BDI, Beck Depression Inventory; BMT, best medical therapy; UNI, unilateral; CANTAB, Cambridge Neuropsychological Test Automated Battery; D-KEFS, Delis-Kaplan Executive function system; DBS, deep brain stimulation; DRS, Depression Rating Scale; DRS-II, dementia rating scale-II; DSM-IV, Diagnostic and Statistical Manual of Mental Disorders, fourth edition; GPi, globus pallidus interna; MDRS, Mattis Dementia Rating Scale; MINI, Mini-international Neuropsychiatric Interview; MMSE, Mini-Mental State Exam; MWCST, Modified Wisconsin Card Sorting Test; NMCST, Nelson Modified Card Sorting Test; NMF, non-motor fluctuations; POMS, Profile of Mood Scale; RAVLT, Rey’s Auditory Verbal Learning Test; RPM, Raven’s Progressive Matrices; SAS, Zung’s Self-rating Anxiety Scale; SAS1, Starkstein Apathy Scale; SCID-II, Structured Clinical Interview for the DSM-III-R Axis II Disorders; SCL-90-R, Self-Report Symptom Inventory 90 Items-Revised; SDS, Zung’s Self-rating Depression Scale; SIP, Sickness Impact Profile (SIP); STAI, State Trait Anxiety Inventory; STN, subthalamic nucleus; UPDRS, Unified Parkinson's Disease Rating Scale; VAMS, Visual Analog Mood Scale; WAIS III, Wechsler Adult Intelligence Scales III.

**Table 3:** DBS effects on apathy and ICDs in patients with PD

*Author*	*Year*	*Outcome measure*	*Type of study*	*No. of patients*	*Main outcome after DBS*
Kim *et al.*^49^	2013	Primary: ICDs MMSE, BDI, modified MIDI	Retrospective	89 STN	ICDs may resolve or improve, or new ones appear after STN DBS. Difference in risk factors for preoperative versus postoperative ICDs.
Lhommée *et al.*^43^	2012	Secondary: Cognition mood, behavior and NMF MDRS, Frontal Score (MWCST, verbal fluency, graphic and motor series) MINI, Ardouin Scale, BDI, BAI and SAS^1^	1-year prospective multicenter	63 STN same cohort as ref. 44	Apathy aggravated. NMF improved. Hyperdopaminergic behaviors and appetitive functioning improved. Same cohort as ref. 44.
Moum *et al.*^48^	2012	Primary: ICDs Chart review using current diagnostic criteria	Retrospective Chart review	159 NA STN or GPi	ICD resolved in 2 of 7 individuals after unilateral or bilateral DBS. 17 patients developed ICD diagnoses postoperatively.
Shotbolt *et al.*^46^	2012	Primary: ICDs Psychiatrist interview HDS, HAS and BDI	17–41 month prospective	29 STN	Pre-surgery: 4 patients had ICDs. All resolved after surgery but 1 recurred after 18 months.
Thobois S *et al.*^51^ See ref. 43	2010	Primary: Apathy and mood Ardouin Scale, BDI, BAI and SAS^1^	1-year prospective multicenter	63 STN	34 patients developed apathy at mean 4.7 (3.3–8.2) months after DBS that was reversible in 17 at 1-year follow-up. 17 developed transient depression after 5.7 (4.7–9.3) months. Apathy, depression and anxiety can occur after surgery as a delayed dopamine withdrawal syndrome.
Ardouin *et al.*^45^	2006	Primary: ICDs: Pathological gambling Systematic open interview by psychiatrist Secondary outcomes: cognition and mood MDRS, SAS^1^ and BDI-II	3-month to 6-year (mean 18 months) retrospective data base analysis multicenter	598 STN	Pathological gambling and other symptoms of the dopamine dysregulation syndrome improved following surgery. Apathy scores tended to increase systematically after surgery and 2 patients had a pathological score on the apathy scale in the long term.
Funkiewitz *et al.*^36^	2004	Primary: cognition, mood and behavior MDRS and WCST Category and literal fluency and graphic and motor series BDI, UPDRS part I	3-year prospective	77 STN	Apathy scores mildly increased. Behavioral changes were rare and transient.

Abbreviations: BAI, Beck Anxiety Inventory; BDI, Beck’s Depression Inventory; DBS, deep brain stimulation; HAS, Hamilton Anxiety Scale; HDS, Hamilton Depression Scale; ICD, impulse control disorders; GPi, globus pallidus interna; MDRS, Mattis Dementia Rating Scale; MIDI, Minnesota Impulsive Disorders Interview; MINI, Mini-International Neuropsychiatric Interview; MMSE, Mini-Mental State Exam; WCST, Wisconsin Card Sorting Test; NMF, non-motor fluctuations; SAS^1^, Starkstein Apathy Scale; STN, subthalamic nucleus; UPDRS, Unified Parkinson's Disease Rating Scale.

**Table 4:** DBS effects on sleep and pain

*Author*	*Year*	*Outcome measure*	*Type of study*	*No. of patients*	*Main outcome after DBS*
Amara *et al.*^60^	2012	Primary: sleep PSQI questionnaire	6 months prospective	53	Unilateral STN DBS improves subjective sleep quality.
Lyons and Pahwa^61^	2006	Primary: sleep Epworth sleepiness Scale	24 months prospective	43	Bilateral STN DBS improved sleep quality. Early morning dystonia and nocturnal akinesia improved. No change in excessive daytime sleepiness.
Hjort *et al.*^59^	2004	Primary: sleep PDSS	3 months prospective	10	Bilateral STN DBS improved sleep quality, but no change in excessive daytime sleepiness or nocturia.
Iranzo *et al.*^58^	2002	Primary: sleep PSQI questionnaire Polysomnography	6 months prospective	11	Subjective and objective improvement in sleep quality (decreased arousal index).
Cury *et al.*^64^	2014	Primary: pain NMSS, Visual Analog Scale	1-year prospective	44	Bilateral STN DBS shows improvement in pain intensity, particularly dystonic and musculoskeletal pain. Motor and non-motor symptoms did not correlate with pain relief.
Kim *et al.*^63^	2012	Primary: pain Clinical Interview	2-year prospective	21	Bilateral STN DBS improves pain, sustained for 24 months. However, *de novo* primarily musculoskeletal pain developed during follow-up.
Gierthmöhlen *et al.*^65^	2010	Primary: pain QST	6 months prospective	17	Bilateral STN DBS shows improvement in pain, however no objective change in pain sensitivity.
Loher *et al.*^62^	2002	Secondary: pain MMS, HDS	12 months prospective	16	Unilateral and bilateral GPi shows improvement in pain at 3-month and 12-month follow-up.

Abbreviations: DBS, deep brain stimulation; HDS, Hamilton Depression Scale; MMS, Mini-Mental Scale; NMSS, Non-Motor Symptoms Scale; PDSS, Parkinson’s Disease Sleep Scale; PSQI, Pittsburgh Sleep Quality Index Questionnaire; QST, quantitative sensory testing; STN, subthalamic nucleus.

**Table 5:** DBS effects on autonomic symptoms in PD

*Author*	*Year*	*Outcome measure*	*Type of study*	*No. of patients*	*Main outcome after STN DBS*
Sumi *et al.*^77^	2012	Primary: cardiovascular symptoms Spectral and transfer function analyses of cardiovascular variability.	Controlled Prospective DBS on/off	41 (28 STN) (13 BMT)	Vagally mediated arterial-cardiac reflex improved post surgery. No difference found between DBS on/off.
Arai *et al.*^70^	2012	Primary: gastrointestinal symptoms^13^ C-acetate breath test.	3 months prospective	16	Improved gastric emptying.
Trachani *et al.*^76^	2012	Primary: cardiovascular symptoms Questionnaire, BP monitoring and neurophysiological tests	6 months prospective	24	No considerable positive or negative effect on ANS cardiovascular regulation, although significant reduction in orthostatic hypotension.
Trachani *et al.*^17^	2010	Primary: sweating Semi-structured questionnaire, Sympathetic skin response	6 months prospective	19	66.7% subjective improvement. No objective improvement.
Winge *et al.*^69^	2007	Primary: urinary symptoms Questionnaires: International Prostate Symptom Score and Danish Prostate Symptom Score Urodynamic investigation	2-year prospective	16	Subjective improvement but no objective improvement found.
Stemper *et al.*^75^	2006	Primary: cardiovascular system Head up tilt testing, (BP), RR intervals (RRI), respiration, and skin blood flow (SBF). Baroreflex sensitivity (BRS)	Cross-sectional DBS on/off	14	Increased peripheral vasoconstriction on stimulation, improving postural hypotension in patients.
Seif *et al.*^67^	2004	Primary: urinary system Urodynamic investigation	Cross-sectional DBS on/off	16	First urge to void and maximum bladder capacity improved significantly in the ON state.

Abbreviations: BP, blood pressure; BMT, best medical therapy; BRS, baroreflex sensitivity; DBS, deep brain stimulation; RRI, RR interval; SBF, respiration and skin blood flow.

**Table 1:** DBS effects on non-motor symptoms as a whole and non-motor fluctuations in PD patients

*Author*	*Year*	*Outcome measure*	*Type of study*	*No. of patients*	*Main outcome after bilateral STN DBS*
Dafsari *et al.*^33^	2016	Primary: NMS as a whole NMSS: 9 domains NMSQuest PDQ-8	Multicenter, open, 6-month prospective study	60	Overall NMSS improved, showing significant improvement in 4 domains: sleep/fatigue, perceptual problems/hallucinations, urinary and miscellaneous.
Wolz *et al.*^24^	2012	Primary: NMS as a whole Semi-structured questionnaire VAS	Cross-sectional study	34	No major immediate effects on frequencies, but improves severity of most NMS, particularly psychiatric symptoms (depression, anxiety, and fatigue).
Reich *et al.*^21^	2011	Primary: NMS as a whole NMSS	3–6 months prospective study	10	Shows holistic and selective beneficial effect on aspects of NMS. Especially on 5 particular domains: sleep, mood/cognition, urinary, sexual, and miscellaneous. Other domains show no change.
Nazzaro *et al.*^22^	2011	Primary: NMS as a whole NMSQuest, PDQ-39	1-year prospective study	24	NMS decreased significantly when assessed with a NMSQuest and significant improvement in QoL.
Zibetti *et al.*^71^	2007	Primary: NMS as a whole Clinical Interview	12–24 months prospective study	36	Ameliorates sleep and constipation.
Ortega-Cubero *et al.*^27^	2013	Primary: NMF Questionnaire	2-year prospective study	20	NMF severity and frequency related to the off-state were reduced.
Witjas *et al.* ^26^	2007	Primary: NMF Questionnaire	1-year prospective study	40	Alleviation of NMF seen, with significant positive effects on sensory, dysautonomic, and cognitive fluctuations. Less consistent in psychic response.

Abbreviations: DBS, deep brain stimulation; NMS, non-motor symptoms; NMF, non-motor fluctuations; NMSS, Non-Motor Symptoms Scale; NMSQuest, non-motor symptoms questionnaire; QoL, quality of life; PD, Parkinson’s disease; VAS, Visual Analog Scale; PDQ, Parkinson’s disease questionnaire.
